# Norovirus Gastroenteritis Outbreak with a Secretor-independent Susceptibility Pattern, Sweden

**DOI:** 10.3201/eid1601.090633

**Published:** 2010-01

**Authors:** Johan Nordgren, Elin Kindberg, Per-Eric Lindgren, Andreas Matussek, Lennart Svensson

**Affiliations:** University of Linköping, Linköping, Sweden (J. Nordgren, E. Kindberg, P.-E. Lindgren, L. Svensson); National Board of Forensic Medicine, Linköping (E. Kindberg); County Hospital Ryhov, Jönköping, Sweden (P.-E. Lindgren, A. Matussek); Capio St Görans Hospital, Stockholm, Sweden (A. Matussek)

**Keywords:** Norovirus, viruses, susceptibility, histo-blood group antigens, GI.3, enteric infections, gastroenteritis, Sweden, research

## Abstract

Norovirus (NoV) is recognized as the commonest cause of acute gastroenteritis among adults. Susceptibility to disease has been associated with histo-blood group antigens and secretor status; nonsecretors are almost completely resistant to disease. We report a foodborne outbreak of GI.3 NoV gastroenteritis that affected 33/83 (40%) persons. Symptomatic disease was as likely to develop in nonsecretors as in secretors (odds ratio [OR] 1.41, 95% confidence interval [CI] 0.46–4.36 vs. OR 0.71, 95% CI 0.23–2.18, p = 0.57). Moreover, no statistical difference in susceptibility was found between persons of different Lewis or ABO phenotypes. The capsid gene of the outbreak strain shares high amino acid homology with the Kashiwa645 GI.3 strain, previously shown to recognize nonsecretor saliva, as well as synthetic Lewis a. This norovirus outbreak affected persons regardless of secretor status or Lewis or ABO phenotypes.

## Abstract 2

Nonsecretors were highly susceptible to norovirus GI.3 in a foodborne outbreak.

Norovirus (NoV) is the leading cause of nonbacterial, acute gastroenteritis among adults and is responsible for numerous outbreaks worldwide (*1*–*4*). The virus is frequently associated with contaminated food, causing >50% of all food-related outbreaks (*5*). Several studies (*6*–*11*) have associated norovirus susceptibility with the presence of an α1,2-linked fucose on histo-blood group antigens (HBGAs), which is determined by the *FUT2* gene (*12*,*13*). Persons carrying >1 functional *FUT2* allele, and thus expressing α1,2 fucosyltransferase 2 (FucT-II), are termed secretor positive (secretors) and can express the A and B blood group antigens as well as H-type 1 and Lewis b (Le^b^) antigens on mucosa and in secretions. Persons lacking FucT-II are termed secretor negative (nonsecretors) and have been shown to be highly protected from infections with several NoV genotypes, including the common GII.4, as well as the Norwalk virus prototype strain (GI.1) (*6*–*11*).

Saliva-binding studies have demonstrated that different NoV strains exhibit different binding patterns (*14*–*16*), with the Norwalk virus (GI.1) mainly recognizing saliva from secretors with blood groups A and O, while exhibiting low or no binding to saliva to nonsecretors and carriers of blood group B, suggesting protection against infection among the latter 2 groups. Virus-like particles (VLPs) of the common GII.4 strains have been found to mainly bind saliva from secretors irrespective of blood group (*16*), although binding to nonsecretor saliva has been described for VLPs of some GII.4 strains (*17*).

Although NoV infections of secretors are well documented (*18*) and a few cases of infected nonsecretors have been reported (*19*,*20*), no virus has been identified in authentic outbreaks that is completely secretor or Lewis antigen independent, where homozygous carriers of the nonsense G428A mutation in *FUT2* are at similar or higher risk for infection than are secretors. We describe a foodborne NoV outbreak in which persons were infected regardless of secretor status or Le phenotypes; and no difference was observed between nonsecretor (Le^a+b−^) persons and secretors regarding risk of symptomatic norovirus infection. Our data provide new knowledge about susceptibility factors and NoV genotypes and suggest that additional studies of host genetic receptor factors and NoV are needed.

## Materials and Methods

### Outbreak Data and Sample Collection

In October 2007, a NoV gastroenteritis outbreak occurred in Jönköping, Sweden, at a seminar for healthcare improvement (October 25–27), attended by 112 healthcare workers from different parts of Sweden. The healthcare workers were asked to take part in this case–control study, and 83 persons, including 4 employees of the restaurant that provided food service, decided to participate. Thirty-three of these 83 persons acquired acute gastroenteritis during or shortly after the seminar. Saliva samples were collected from all 83 participants in the study and stored at –20°C until further use. Stool samples (n = 4) were obtained from the cook, 2 employees, and 1 participant of the conference with symptoms of NoV gastroenteritis. Epidemiologic investigations indicated that the lunch on the first day was contaminated with NoV and was subsequently the cause of the outbreak. The cook was ill 4 days before the outbreak started, and 3 days later other employees of the restaurant became ill, suggesting the restaurant employees as the probable source of NoV contamination in the food. NoV disease was identified by at least 1 of the following signs or symptoms: vomiting, diarrhea, or nausea combined with stomach ache ≈12–60 hours after ingesting the meal. Description of symptoms was obtained through a questionnaire sent to all participants in the study. The study was approved by the local ethics committee (M205-04 T48-08).

### DNA Extraction from Saliva

Genomic DNA from 200 μL saliva was extracted by using QIAamp DNA Mini Kit (QIAGEN, Hilden, Germany) according to the instructions of the manufacturer (Blood and Body Fluid Spin Protocol). Extracted DNA was stored in AE buffer (QIAGEN) at –20°C until PCR amplification.

### PCR Amplification of *FUT2* and Determination of *FUT2* 428 Genotype

The *FUT2* gene amplification by PCR was performed as previously described (*6*). Genotyping of the G428A mutation in the *FUT2* gene was performed as previously described (*6*,*7*,*21*). These methods can distinguish between carriers of the homozygous wild-type, heterozygous, and homozygous mutated genotype.

### Detection of Histo-Blood Group Antigens in Saliva

The ABO histo-blood group phenotype of secretor-positive persons and the Lewis phenotype of all 83 persons were determined by a saliva-based ELISA, essentially as described by Bucardo et al. (*6*) and Rydell et al. (*22*). Protein concentration was determined in boiled (5 min) and centrifuged (5 min, 10,000 rpm) saliva by means of a Bradford assay. ELISA plates (NUNC 96F Maxisorp; Thermo Fisher Scientific, Roskilde, Denmark) were coated with saliva, diluted to a final protein concentration of 1 μg/mL in coating buffer (0.1 M carbonate–bicarbonate buffer, pH 9.6); plates were incubated for 2 h at 37°C followed by 4°C overnight. The following day, the plates were washed 4 times with washing buffer (0.9% NaCl, 0.05% Tween 20 [Sigma-Aldrich, St. Louis, MO, USA]), and then incubated for 1.5 h at 37°C with antibodies α-A (ABO1 clone 9113D10), α-B (ABO2 clone 9621A8) (Diagast, Loos Cedex, France), α-Le^a^ (Seraclone, LE1 clone 78FR 2.3), and α-Le^b^ (Seraclone LE2 clones LM129-181 and 96 FR2.10) (Biotest AG, Dreieich, Germany). Antibodies were diluted 1:5000 in phosphate-buffered saline with 10% fetal bovine serum (Invitrogen AB, Lidingö, Sweden) and 0.05% Tween 20 (Sigma-Aldrich). After 4 washes, horseradish peroxidase–conjugated goat anti-mouse IgG (heavy plus light chain) (Bio-Rad Laboratories, Hercules, CA, USA), diluted 1:7,500, was added, and plates were incubated for another 1.5 h at 37°C and subjected to 4 final washes. The reaction was developed using 3′,3′,5′,5-tetramethylbenzidine (DakoCytomation, Carpinteria, CA, USA) and stopped by addition of 2M H_2_SO_4_. The plate was read at 450 nm in a spectrophotometer. The cutoff value was twice the mean level of 6 known negative samples. The α-Le^b^ antibody cross-reacted weakly with Le^a^; this signal was subtracted from the Le^b^ values read in Le^a^-positive persons.

### Virus RNA Extraction and Reverse Transcription

RNA extraction from the 4 collected stool specimens was performed by using the EZ1 robot (QIAGEN) according to the manufacturer’s instructions and stored at –80°C until used for reverse transcription. Reverse transcription was performed as previously described (*6*,*23*), by using random hexamer primers (GE Healthcare, Uppsala, Sweden) and Illustra Ready-To-Go RT-PCR beads (GE Healthcare).

### Norovirus Detection with Real-Time PCR

NoV detection and quantification were performed with a real-time PCR specific for the open reading frame (ORF) 1–ORF2 junction, as described by Nordgren et al. (*24*). This real-time PCR assay can semiquantify and distinguish between NoVs GI and GII (*24*). PCR amplification of the N-terminal and shell (N/S) region was performed on a PTC-100TM thermal cycler (MJ Research Inc., South San Francisco, CA, USA) in a 50-μL mixture composed of 1.33 U of Expand High Fidelity polymerase (Boehringer Mannheim GmbH, Mannheim, Germany), 5 μL of the supplied buffer (including 1.5 mmol/L MgCl_2_; Boehringer Mannheim GmbH), 100 µM GeneAmp dNTP mixture with dTTP (Applied Biosystems, Branchburg, NJ, USA), 200 nM forward primer NVG1f1b (5′-CGY TGG ATG CGN TTC CAT GA-3′) (*24*), 200 nM reverse primer G1SKR (5′-CCA ACC CAR CCA TTR TAC A-3′) (*25*), and 5 μL template DNA.

### Nucleotide Sequencing of the Norovirus N/S Region and Virus Genotyping

Nucleotide sequencing of the N/S region was performed by Macrogen Inc. (Seoul, South Korea). The sequencing reaction was based on BigDye chemistry; NVG1f1b forward primer (*24*) and G1SKR reverse primer (*25*) were used as sequencing primers. The amplicons were sequenced twice in each direction. Sequence alignment of the Jönköping (JKPG) strain and reference NoV genotypes was performed by using the ClustalW algorithm, version 1.8 (www.ebi.ac.uk/clustalw), with default parameters, on the European Bioinformatics Institute server. We performed phylogenetic analysis using the MEGA 4.0 software package (www.megasoftware.net), and the phylogenetic tree was constructed using the neighbor-joining and Kimura 2-parameter methods. Significance of the taxonomic relationships was obtained by bootstrap resampling analysis (1,000 replications). Assignment of genotypes used reference strains described by Zheng et al. (*26*).

### PCR Amplification of the Norovirus Capsid Gene

To amplify the gene encoding the NoV capsid, we set up a PCR mixture containing 2.5 μL 10× native *Pyrococcus furiosus* (*pfu*) polymerase buffer (Invitrogen AB, Lidingö, Sweden), 200 µM GeneAmp dNTP mix with dTTP (Applied Biosystems), 200 nM forward primer CapGI3fw (5′-GAT CTC CTG CCC GAT TAT GTA AAT GAT GAT G-3′, this study), targeting the end of ORF1 and beginning of ORF2, 200 nM reverse primer CapGI3rv (5′-CAT TAT GAT CTC CTA ATT CCA AGC CTA CGA GC-3′, this study), specific for the end of ORF2 and beginning of ORF3, 5 μL cDNA, 2.5 U native *pfu* DNA polymerase (Stratagene, La Jolla, CA, USA), and 36 μL RNAse-free water. After initial denaturation at 94°C for 5 min, PCR amplification was performed with 40 cycles of 94ºC for 1 min, 58ºC for 1 min, and 72ºC for 2 min, and thereafter a final elongation at 72ºC for 10 min. The PCR products were visualized by electrophoresis on a 2% agarose gel, using staining with ethidium bromide and UV transillumination.

### Cloning of the Norovirus Capsid Gene and Nucleotide Sequencing

The capsid fragment was cloned into a pPCR-Script Amp SK(+) vector and transformed into XL10-Gold Kan ultracompetent cells, using the Stratagene PCR-Script Amp Cloning Kit (Stratagene) according to the manufacturer’s instructions. After overnight incubation of 2 separate colonies from each transformation reaction, plasmid DNA was extracted and purified, using the Plasmid Miniprep Kit (QIAGEN) according to the manufacturer's instructions. Nucleotide sequencing was performed on 2 separate plasmid extractions from each sample (n = 2) by Macrogen Inc., by using the BigDye chemistry with M13 forward and reverse primers. The nucleotide sequences for the N/S region or the complete capsid gene of the JKPG isolates are available under GenBank accession nos. FJ711163, FJ711164, and FJ711165.

### Statistical Analysis

Categorical data were analyzed using the Fisher exact test with 2-tailed significance. Unadjusted odds ratios (ORs) and 95% confidence intervals (CIs) were calculated using SPSS 14.0 for Mac (SPSS Inc., Chicago, IL, USA).

## Results

### Outbreak Description

**Figure 1:**
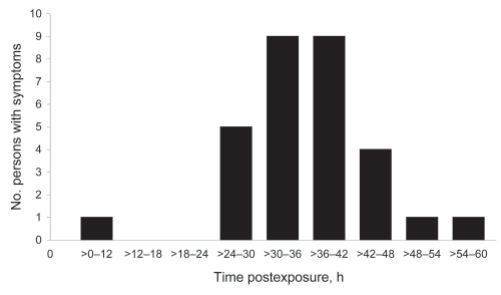
Time to onset of symptoms after patient exposure to norovirus-contaminated food (n = 30), Sweden. Zero indicates the time point for serving and ingesting the contaminated food.

A total of 83 persons responded to the questionnaire and participated in the study. Among them, 33 (40%) were symptomatic, and 50 (60%) reported no symptoms. The latter group may include exposed asymptomatic as well as nonexposed persons. The onset of symptoms varied from 1 through 3 days (mean 36 h) after ingestion of the contaminated meal (Figure 1); mean duration of symptoms was 35 h. The most common symptoms were vomiting (23/32, 72%), diarrhea (20/32, 63%), joint pain (18/32, 56%), and headache (14/32, 44%). Most symptomatic persons (n = 30) had diarrhea, vomiting, or both, whereas the remaining 3 persons had nausea and stomach ache.

### Distribution of ABO and Lewis Phenotypes and Secretor Status

To investigate whether persons associated with the outbreak had a skewed HBGA profile, we determined the ABO, Lewis, and secretor status distributions among symptomatic and asymptomatic/nonexposed persons and compared them with results from earlier investigations of the population in Sweden (Table 1). The ABO, Lewis, and secretor status distributions were in the normal ranges compared with those investigations (*21*,*27*) (Table 1), with the exception of the AB and Lewis negative phenotypes. Furthermore, we observed that all HBGAs investigated, except AB (n = 1), were found among asymptomatic/nonexposed and symptomatic persons. Sixty-one persons were secretor and Lewis positive; of these, 52 (85%) were positive for Le^a^ and Le^b^ in saliva. The 4 persons from whom NoV was isolated were all secretors, having ALe^a−b+^, ALe^a−b+^, OLe^a−b−^, and OLe^a−b+^ HBGA profiles, respectively.

**Table 1:** Distribution of histo-blood group antigens phenotypes and secretor status among 83 participants in a case–control study of a norovirus gastroenteritis outbreak in Sweden, 2007*

Antigen and secretor status	No. (%) symptomatic persons, n = 33	No. (%) asymptomatic/ nonexposed persons, n = 50†	Total no. (%) persons, n = 83	Normal distribution, % (95% CI)†‡
Blood type (n = 68)				
A	14 (44)	18 (56)	32 (47)	47 (37–56)
B	2 (17)	10 (83)	12 (18)	15 (8–22)
O	10 (43)	13 (57)	23 (34)	33 (23–40)
AB	0	1 (100)	1 (1)	7 (2–12)
Lewis (n = 83)				
Le^a+b–^	7 (58)	5 (42)	12 (14)	18 (11–26)
Le^a–b+^	23 (38)	38 (62)	61 (73)	76 (68–84)
Le^a–b–^	3 (30)	7 (70)	10 (12)	6 (1–10)
Secretor status (n = 83)				
Secretor§	26 (38)	42 (62)	68 (82)	80 (72–88)
Nonsecretor	7 (47)	8 (53)	15 (18)	20 (12–28)

*ABO blood group could only be determined for the 68 secretor-positive persons (26 symptomatic and 42 asymptomatic). CI, confidence interval. †p>0.15 ‡See Larsson et al. (*27*). §Homozygous and heterozygous.

### Difference in Susceptibility to Symptomatic Infection between Secretors and Nonsecretors

Previous studies have shown a strong correlation between symptomatic NoV infections and the secretor-positive phenotype (*7*–*10*). To investigate whether secretor and Lewis status were associated with susceptibility in this study, secretor and Lewis status were determined by genotyping and phenotyping of all persons. We observed that 7/15 (47%) nonsecretors were symptomatically infected, compared with 26/68 (38%) secretors (Table 1). Although the calculated OR for nonsecretors was ≈2× that of secretors (OR 1.41 vs. 0.71), the differences were not significant (Table 2). Thus, in the group studied, nonsecretors were as likely as secretors to be symptomatically infected by norovirus. The same pattern was observed for the Lewis phenotypes; no statistical difference was found between persons with Le^a+b−^, Le^a−b+^, or Le^a–b–^ regarding risk for symptomatic infection (Table 2). None of the Lewis-negative nonsecretors (n = 3) were symptomatically infected. The *FUT2* G428A genotyping did not show any significant differences between heterozygous secretors and homozygous secretors (OR 1.20, 95% CI 0.49–2.95 vs OR 0.67, 95% CI 0.27–1.65; Table 2).

**Table 2:** Influence of secretor status, *FUT2* polymorphism, and histo-blood group antigens on risk for norovirus GI.3 symptomatic infection, Sweden*

Secretor status	OR (95% CI)	p value
Secretor, n = 68	0.71 (0.23–2.18)	0.57
Nonsecretor, n = 15	1.41 (0.46-4.36)	0.57
*FUT2* 428 polymorphism		
G/G (secretor), n = 35	0.67 (0.27–1.65)	0.50
G/A (secretor), n = 33	1.20 (0.49–2.95)	0.82
A/A (nonsecretor), n = 15	1.41 (0.46–4.36)	0.57
Histo-blood group antigens		
Blood type,† n = 68		
A, n = 32	1.56 (0.58–4.16)	0.46
B, n = 12	0.27 (0.05–1.33)	0.11
O, n = 23	1.39 (0.50–3.89)	0.60
AB, n = 1	Not applicable‡	1.0
Lewis, n = 83		
Le^a+b–^, n = 12	2.42 (0.70–8.42)	0.21
Le^a-b+^, n = 61	0.73 (0.27–1.95)	0.61
Le^a–b–^, n = 10	0.61 (0.15–2.57)	0.73

*FUT, fucosyltransferase; OR, odds ratio; CI, confidence interval. †Compared between secretors. ABO blood group could only be determined for the 68 secretor-positive persons. ‡No carrier of blood type AB was symptomatically infected with norovirus.

### Association between ABO Blood Types and Symptomatic Infection

Previous studies have shown that ABO blood types are associated with susceptibility to symptomatic NoV infections, with persons having blood type B at lower risk for infection when challenged with Norwalk virus (GI.1) (*8*,*28*). In this outbreak, we found that symptoms developed in 2/12 (17%) of persons with blood group B (Table 1). Although persons with blood group B were infected to a lesser extent than persons with other blood groups, this reduction was not significant (OR 0.27, 95% CI 0.05–1.33; Table 2). FFurthermore, no significant differences were found when comparing symptomatic and nonsymptomatic persons with blood types A and O (OR 1.56, 95% CI 0.58–4.16, and OR 1.39, 95% CI 0.50–3.89, respectively) (Table 2). Thus, no blood type provided complete protection or was associated with a higher or lower risk for disease.

### Association between Blood Type, Secretor Status, and Clinical Symptoms

A recent study suggested that blood type can have an influence on clinical symptoms after NoV infection (*29*). To investigate whether this would apply in this outbreak, blood types, secretor status, and clinical symptoms were compared. We did not find any correlation between blood type and secretor status with clinical symptoms (Table 3).

**Table 3:** Relationship between clinical symptoms of norovirus infection and secretor status and blood type distribution among 83 participants in a case–control study of a norovirus gastroenteritis outbreak in Sweden, 2007*

Data	No. (%) persons reporting symptom			
	Diarrhea	Vomiting	Joint pain	Headache
Blood type				
A, n = 14	9 (64)	12 (86)	9 (64)	7 (50)
B, n = 2†	1 (50)	0 (0)	0 (0)	0(0)
O, n = 9‡	6 (67)	6 (67)	4 (44)	5 (56)
Secretor, n = 25‡	16 (64)	18 (72)	13 (52)	12 (48)
Nonsecretor, n = 7	4 (57)	5 (71)	5 (71)	2 (29)
Total	20 (63)	23 (72)	18 (56)	14 (44)

*HBGA, histo-blood group antigen. †One person with HBGA type B experienced only nausea and stomachache. ‡One secretor-positive person with HBGA type O did not provide a description of symptoms.

### Similarity of JKPG and Kashiwa645 Strains in the P2 Domain and Putative Receptor Binding Sites

**Figure 2:**
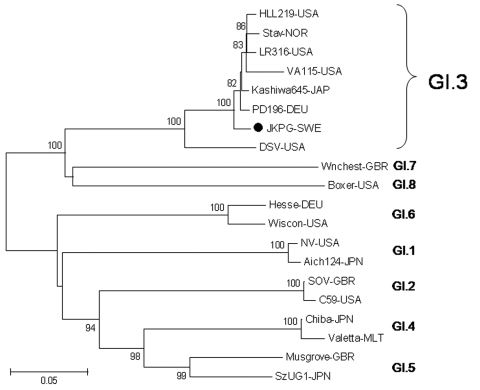
Phylogenetic analysis of amino acids of the norovirus capsid gene from the gastroenteritis outbreak in Jönköping, Sweden (JKPG, •) and reference strains. The tree was constructed using the neighbor-joining and Poisson correction methods, with MEGA 4.0 software (www.megasoftware.net). Bootstrap values are shown at the branch nodes (values <70% are not shown). Reference sequences were collected from Genbank and represent the 8 genotypes of GI as described by Zheng et al. (*26*). Scale bar indicates nucleotide substitutions per site. NV-USA [M87661], Aich124-JPN [AB031013], SOV-GBR [L07418], C59-USA [AF435807], HLL219-USA [AF414403], Stav-Nor [AF145709], LR316-USA [AF414405], VA115-USA [AY038598], Kashiwa645-JAP [BD011871], PD196-DEU [AF439267], JKPG-SWE [FJ711163] DSV-USA [U04469], Chiba-JPN [AB042808], Valetta-MLT [AJ277616], Musgrove-GBR [AJ277614], SzUG1-JPN [AB039774], Hesse-DEU [AF093797], Wiscon-USA [AY502008], Wnchest-GBR [AJ277609], Boxer-USA [AF538679].

NoV GI was detected by real-time PCR in all collected stool specimens (n = 4); three of these isolates (881–883) were subsequently genotyped by nucleotide sequencing of the N/S region. The fourth sample could not be genotyped because of low virus concentration in the stool sample. Phylogenetic analysis clustered the 3 isolates with NoV GI.3 strains (data not shown). The entire capsid gene was subsequently sequenced from 2 isolates and compared with reference strains (Figure 2). The closest amino acid similarity (98.0%) of the complete capsid gene was found with strain PD196-DEU (GI.3), isolated in Germany 2000, and with the Kashiwa645 (GI.3) strain (97.8%), used in an earlier VLP binding study (*14*).

We then investigated the amino acid composition of the capsid P2 domain of the outbreak strain and compared it with the Kashiwa645 and Norwalk strains. Although the JKPG strain differed by 4 aa at positions 344, 367, 377, and 397 (97.1% homology) compared with Kashiwa645, it shared only ≈50% aa positions with the GI.1 Norwalk strain.

## Discussion

Previous studies have shown a strong (*6*–*11*) but not absolute (*19*,*20*) association between nonsecretors and protection from symptomatic NoV disease. In contrast to these observations, we report a foodborne NoV outbreak affecting persons regardless of secretor, Lewis, or ABO phenotype.

Because the host genetic observation of this outbreak was unexpected, attempts were made to compare the HBGA frequencies of the participating persons with those of the population in Sweden. The ABO, secretor, and Lewis phenotype frequencies in this study agreed with earlier investigations from the population in Sweden (*21*,*27*) (Table 1), with the exception of the AB and Lewis negative phenotypes, probably due to their low prevalence in combination with the small sampling set. Seven (8%) secretor and 3 (4%) nonsecretor persons were Lewis negative and hence lacked Lewis antigen in saliva. Genotyping of the *FUT2* G428A nonsense mutation confirmed secretor-negative genotype of all Le^a+b–^ persons and the secretor-positive genotype of Le^a−b+^ persons.

Comparison of secretor and Lewis phenotypes regarding susceptibility to symptomatic NoV infection showed that nonsecretors were as susceptible to symptomatic disease as secretors. Consistent with the lack of secretor association, no significant difference in susceptibility was noted between Le^a+b−^ and Le^a−b+^ persons (OR 2.42, 95% CI 0.70–8.42 vs. OR 0.73, 95% CI 0.27–1.95). None of the nonsecretors who were also Lewis negative (n = 3), hence lacking the Le^a^ antigen and ABO in saliva, were symptomatically infected. These findings indicate but do not prove that the Le^a^ antigen is a putative receptor for this norovirus strain. The disease pattern of this outbreak is consistent with the findings by Shirato et al. (*14*), who observed strong binding to synthetic Le^a^ and saliva from secretors and nonsecretors with VLPs from the GI.3 Kashiwa645 strain, which shares high homology with the JKPG strain in the P2 domain. A mechanistic virus–saliva binding study with the authentic virus would have been desirable, but limited amounts of virus restricted our attempts to investigate if the outbreak virus binds to saliva both from secretors and nonsecretors.

No ABO phenotype provided protection or was associated with a higher risk of disease, although persons with blood type B exhibited a low (17%) frequency of symptomatic infection (Tables 1, 2). Blood type B has previously been associated with protection from disease when challenged with the Norwalk virus (*28*) and was also supported by in vitro binding studies with VLPs (*16*). Shirato et al. (*14*) found that the Kashiwa645 (GI.3) VLP bound weaker to saliva from blood type B-positive persons compared with types A and O, which agrees with the disease pattern observed in this outbreak. It is possible that the α-gal in the blood type B structure partly covers an epitope needed for binding and hence decreases the ability of the JKPG strain to infect carriers of blood type B.

One limitation of our study is that some of the asymptomatic persons may not have been exposed to the virus. This possibility could result in sampling artifacts because symptoms could have developed in the unexposed (and thus seemingly protected) persons if they had been exposed. However, this possibility is unlikely to influence the main findings of this study, namely the secretor- and HBGA-independent infection pattern, because symptomatic persons were found in all HBGA groups.

An advantage with genotyping compared with phenotyping is that the roles of heterozygosity and homozygosity in disease susceptibility can be investigated. In this study, we did not observe any significant differences between heterozygous and homozygous secretors (OR 1.20, 95% CI 0.49–2.95 vs. OR 0.67, 95% CI 0.27–1.65) (Table 2), which is in agreement with earlier observations (*7*,*9*).

By comparing our strain with a reference strain of the same cluster (Kashiwa645) used in earlier binding studies (*14*), we aimed to elucidate structural similarities or differences that could explain the unique disease profile of the outbreak. The JKPG strain investigated in this outbreak shares high amino acid homology with the GI.3 Kashiwa645 strain (Figure 2). Shirato et al. (*14*) found that the Kashiwa645 strain bound to the same extent to secretor and nonsecretor saliva. However, another consideration is that Asian nonsecretors in the study (*14*) were identified as carriers of a missense mutation at nt 385 (A→T) and thus are incomplete nonsecretors, producing small amounts of ABO and Le^b^ HBGA in secretions. The similarities between the binding profile of the Kashiwa645 strain and the disease profile of the JKPG strain indicate that saliva binding may be used to assess susceptibility patterns for individual NoV strains.

In conclusion, we report a foodborne NoV outbreak infecting persons irrespective of Lewis and secretor status, with Le^a+b−^ persons homozygous for the *FUT2* G428A nonsense allele being symptomatically infected at similar rates compared with secretors. Our observed disease pattern is in concordance with saliva binding specificities of VLP based on the Kashiwa645 strain, sharing high homology in the P2 domain with the JKPG strain. Increased knowledge of susceptibility factors for norovirus disease will be helpful in the development of preventive or therapeutic measures for infection.
